# New Diterpenoids from Soft Coral *Sarcophyton ehrenbergi*

**DOI:** 10.3390/md11114318

**Published:** 2013-10-30

**Authors:** Shang-Kwei Wang, Mu-Keng Hsieh, Chang-Yih Duh

**Affiliations:** 1 Asia-Pacific Ocean Research Center, National Sun Yat-sen University, Kaohsiung 804, Taiwan; E-Mail: skwang@cc.kmu.edu.tw; 2 Department of Microbiology, Kaohsiung Medical University, Kaohsiung 807, Taiwan; 3 Department of Marine Biotechnology and Resources, National Sun Yat-sen University, Kaohsiung 804, Taiwan

**Keywords:** *Sarcophyton ehrenbergi*, diterpenoids, cytotoxicity, anti-HCMV

## Abstract

Continuing chemical investigation on the acetone extracts of the soft coral *Sarcophyton ehrenbergi* collected off the coast of San-hsian-tai, Taitong County, Taiwan led to the isolation of two new diterpenoids, ehrenbergol C and acetyl ehrenberoxide B (**1** and **2**). The structures of these isolated metabolites were elucidated through extensive spectroscopic analyses. Moreover, *in vitro* tests show that compounds **1** and **2** displayed antiviral activity towards human cytomegalovirus, with EC_50_ of 20 and 8.0 µg/mL, respectively.

## 1. Introduction

Marine organisms, which have developed unique metabolic and physiological capabilities to ensure survival in extreme marine habitats, offer the potential to produce novel bioactive secondary metabolites that would not be produced by terrestrial organisms [1]. Soft corals of the genus *Sarcophyton* have been reported as a rich source of diterpenoids [1]. These constituents, mainly macrocyclic cembranes and their derivatives, represent important chemical defense substances for the animals against their natural predators [2]. Cembranoids have been previously reported to exhibit a range of biological activities including antitumor [3,4,5,6,7,8,9], ichthyotoxic [10], anti-inflammatory [11], neuroprotective [12], antibacterial [13], antiangiogenic [14], antimetastatic [14], anti-osteoporotic [15], cytotoxic [16,17,18] and antiviral properties [19,20].

**Figure 1:**
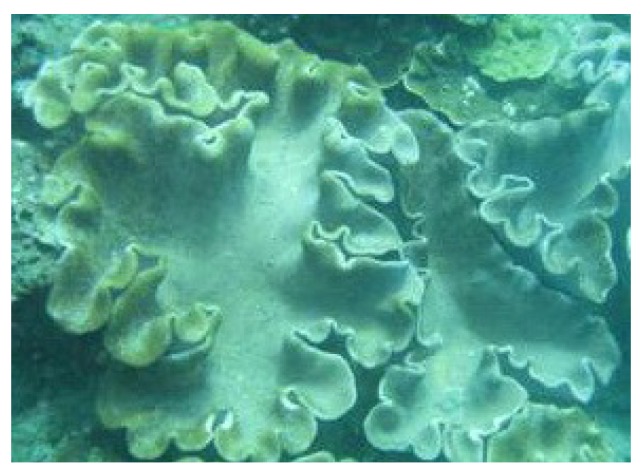
Soft coral *Sarcophyton* *ehrenbergi*.

**Figure 2:**
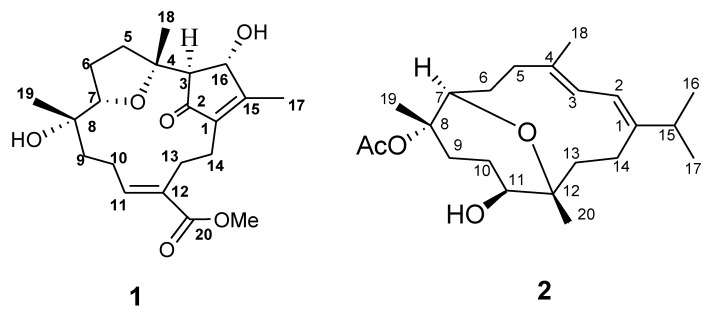
Structures of compounds **1** and **2**.

Fifteen cembranoids were previously reported from the Taiwanese soft coral *Sarcophyton ehrenbergi* [19,20,21]. Continuing chemical investigation of the soft coral *S. ehrenbergi* (Figure 1) collected at San-Hsian-Tai (Taitong County, Taiwan) resulted in the isolation of two new diterpenoids, designated as ehrenbergol C and acetyl ehrenberoxide B (**1** and **2**) (Figure 2). Herein, we describe the purification, structure elucidation, cytotoxicity and antiviral evaluation of these metabolites.

## 2. Results and Discussion

Compound **1** was isolated as a colorless oil, [α]^25^_D_ +95.0 (*c* 0.2, CHCl_3_). The IR spectrum of **1** exhibited absorptions due to hydroxyl (3444 cm^−1^) and conjugated enone (1696 cm^−1^) functionalities. The presence of the conjugated enone was also confirmed by the UV spectrum [λ_max_ (log ε) 223 nm (3.42)]. HRESIMS exhibited a pseudo molecular ion peak at *m/z* 401.1939 [M + Na]^+^, consistent with the molecular formula of C_21_H_30_O_6_.

The structure of **1** was solved by a combination of 1D and 2D NMR methods. The resonances at *δ*_C_ 205.9 (qC), 141.4 (qC), and 168.9 (qC), in the ^13^C NMR and DEPT spectra suggested the presence of a tetrasubstituted conjugated enone (Table 1). Furthermore, the presence of four oxygenated carbons was inferred from the carbon signals at *δ*_C_ 73.9 (qC), 83.2 (qC), 85.6 (CH), and 75.4 (CH). NMR spectroscopic data [*δ*_H_ 6.76 (1H, t, *J* = 7.2 Hz) and 3.75 (3H, s); *δ*_C_ 168.2 (C, C-20), 130.7 (C, C-12), 144.7 (CH, C-11), and 51.6 (CH_3_, COOMe)] revealed the presence of an α,β-unsaturated methyl ester functionality. Six methylene groups were deduced from six triplet signals at *δ*_C_ 36.6, 34.3, 28.5, 24.3, 23.8, and 21.8, a methine signal at *δ*_C_ 60.8, and, finally, three methyl signals at *δ*_C_ 13.4, 28.4, and 24.2.

**Table 1:** NMR data for compound **1**.

Position	*δ*_H_^a^ (*J* in Hz)	*δ*_C_^b^, type	HMBC	COSY	NOESY
1		141.4, qC			
2		205.9, qC			
3	2.13, d (2.0)	60.8, CH	2, 4, 16	16, 18	5a
4		83.2, qC			
5a	3.11, ddd (12.0, 8.8, 3.2)	34.3, CH_2_		6b, 18	3
5b	1.67 m		6		18
6a	1.56, m	28.5, CH_2_		7	
6b	1.84, m		7	5a, 7	7
7	3.82, dd (10.4, 6.0)	85.6, CH	8, 9	6a, 6b	6b, 19
8		73.9, qC			
9a	1.59, m	36.6, CH_2_	8, 10, 11		
9b	1.45, m		8, 10, 11	10	
10a	1.98, m			9b, 11	13b
10b	1.99, m	23.8, CH_2_	9, 12	9b, 11	11, 19
11	6.76, t (7.2)	144.7, CH	9, 10, 20	10	10b
12		130.7, qC			
13a	2.46, m	24.3, CH_2_	11, 12, 14, 20	14	
13b	2.63, m		1, 11, 12, 14, 20	14	14a
14a	2.46, m	21.8, CH_2_	1, 2, 13, 15	13	13b
14b	2.47, m			13	17
15		168.9, qC			
16	4.92, d (6.0)	75.4, CH	1, 4, 15	3, 17	17, 18
17	1.94, s	13.4, CH_3_	1, 15, 16	16	14b, 16
18	1.42, s	28.4, CH_3_	3, 4, 5	5a	7, 5b, 16
19	1.09, s	24.2, CH_3_	7, 8, 9		6a, 7, 10b
20		168.2, qC			
OMe	3.75, s	51.6, CH_3_	20		

^a^ Spectra were measured in CDCl_3_ (400 MHz); ^b^ Spectra were measured in CDCl_3_ (100 MHz).

**Figure 3:**
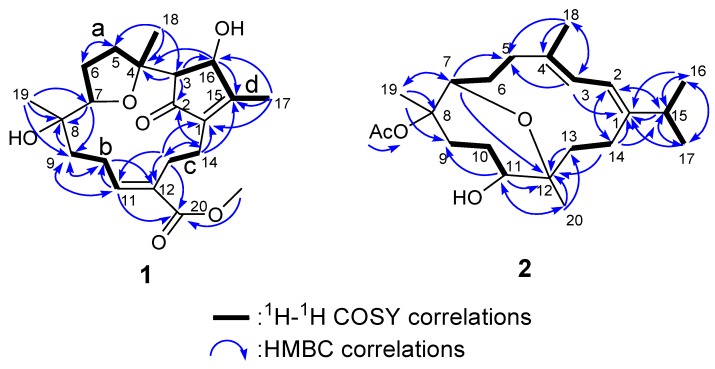
COSY and HMBC correlations of compounds **1** and **2**.

The combined use of ^1^H–^1^H COSY and HMQC on **1** allowed us to distinguish four spin systems (Figure 3a–d). A HMBC experiment was used to assemble the skeletal fragments through quaternary carbons and heteroatoms. Thus, these substructures were connected through HMBC correlations between the protons H_2_-14 (*δ*_H_ 2.46) and the carbons C-1 (*δ*_C_ 141.4), C-2 (*δ*_C_ 205.9), C-15 (*δ*_C_ 168.9), and C-12 (*δ*_C_ 130.7), between the methine proton H-3 (*δ*_H_ 2.13) and carbon C-2, between the methyl protons Me-19 and the carbon C-7, C-8 and C-9, between the methyl protons Me-18 (*δ*_H_ 1.42) and carbons C-3 (*δ*_C_ 60.8), C-4 (*δ*_C_ 83.2), and C-5 (*δ*_C_ 34.3), and between H_2_-13/H-11/OMe-20 and the carbon C-20. These relationships are represented in Figure 3.

**Figure 4:**
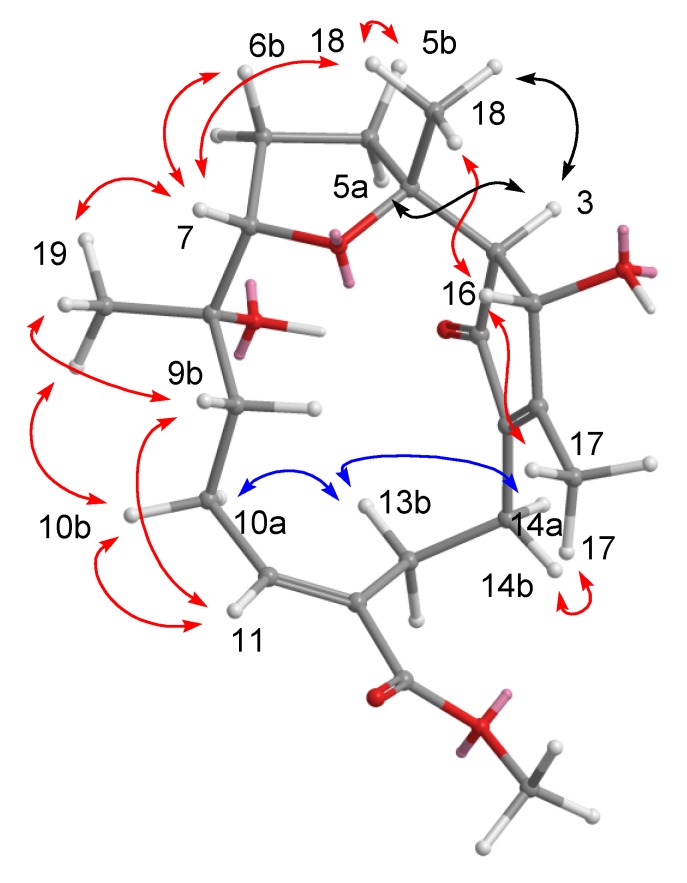
NOESY correlations of compound **1**.

All these data allowed us to identify compound **1** having the same planar framework as lobocrasol isolated from soft coral *Lobophytum crassum* [22]. With the gross structure of **1** in hand, the relative stereochemistry of compound **1** was deduced from NOESY correlations and Chem3D Ultra 9.0 (Figure 4). The *Z* geometry of the Δ^11^ double bond was established by the NOESY correlation observed between H-11 and H-10b and between H-10a and H-13b. NOESY correlations between H-7/H_3_-19, H-7/H_3_-18, and H_3_-18/H-3 indicated that these protons are on the same face of the ring system, thereby establishing the relative configuration of **1**. The relative stereochemistry of C-7 and C-8 were different from lobocrasol; however, the absolute structure was not determined due to the limited amount of the sample.

**Figure 5:**
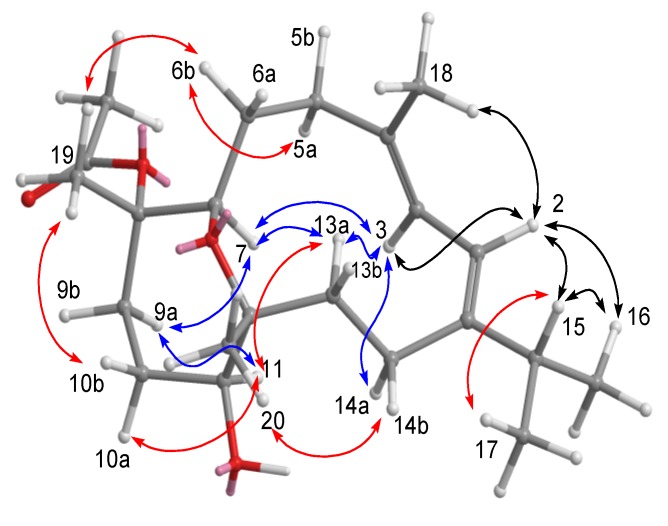
NOESY correlations of compound **2**.

Compound **2** analyzed for C_2__2_H_3__6_O_4_ from HRESIMS and ^13^C NMR spectroscopic data (Table 2), corresponding to five degrees of unsaturation. The IR spectrum of **2** at 3445 cm^−1^ demonstrated a broad absorption band diagnostic of hydroxy group. The presence of one oxygenated methine [*δ*_H_ 4.05 (t, 1H, *J* = 3.2 Hz) and *δ*_C_ 75.3 (C-7)] and an oxygenated quaternary carbon [*δ*_C_ 80.4 (C-12)] implied that an oxygen bridge is probably present between C-7 and C-12, which was supported by the HMBC correlations from H-7 to C-12. The NMR spectroscopic data (Table 2) indicated that **2** possesses an acetoxy [*δ*_H_ 1.66 (3H, s); *δ*_C_ 169.8, 22.1] and a conjugated diene [*δ*_H_ 6.05 (1H, d, *J* = 8.4 Hz) and 6.37 (1H, d, *J* = 8.4 Hz); *δ*_C_ 151.0 (C, C-1), 117.9 (CH, C-2), 123.5 (CH, C-3), and 132.6 (C, C-4)]. The above functionalities suggest that metabolite **2** must consist of a 14-membered ring diterpenoid incorporating an oxepane ring, a hydroxy, an acetoxy and a conjugated diene. Correlations deduced from extensive analyses of the ^1^H–^1^H COSY correlations of **2** in C_6_D_6_ enabled initially the establishment of five partial structures. The structural fragments were subsequently interconnected by the HMBC correlations (Figure 3). Two oxygen bearing carbons at *δ*_C_ 87.0 (C) and 78.3 (CH) were ascribable to C-8 and C-11 on the basis of the HMBC correlations from Me-19 to C-7, C-8, and C-9 and from Me-20 to C-11, C-12, and C-13. The attachment of isopropyl to C-1 was established on the grounds of HMBC correlations from Me-16/Me-17 to C-15 and C-1. The positions of the conjugated double bonds at C-1/C-2 and C-3/C-4 were confirmed by the HMBC cross-peaks from Me-18 to C-3, C-4, and C-5, as well as a COSY correlation between H-2 and H-3. The planar structure of compound **2** was thus elucidated. The relative configuration and the detailed ^1^H NMR spectroscopic data assignments of **2** were determined mainly by the assistance of the NOESY experiment (Figure 5). The crucial NOE correlations between H-2/H-3, H-2/Me-16, H-2/Me-18, H-2/H-15, H-3/H-7 (*δ*_H_ 4.05), and H-3/H-14a (*δ*_H_ 2.64) indicated that the geometries of the two olefins at C-1/C-2 and C-3/C-4 were assigned as both *E*. The coupling constant between H-2 and H-3 (*J*_2,3_ = 8.4 Hz) [23] further suggested the *s-trans* geometry of the conjugated double bonds. Furthermore, the crucial NOE correlations between H-7/H-9a (*δ*_H_ 2.13), H-11/H-9a, H-11/H-10a (*δ*_H_ 1.74), H-11/H-13a (*δ*_H_ 1.97), Me-19/H-6b (*δ*_H_ 1.84), Me-19/H-10b (*δ*_H_ 1.78), Me-20/H-14b, Me-20/H-10b, and H-3/H-14a (*δ*_H_ 2.64) demonstrated the 7R*, 8S*, 11S*, and 12R* configurations as depicted in Figure 5. Accordingly, the structure of **2** was determined as (7R*,8S*,11S*,12R*,1*Z*,3*E*)-8-acetoxy-11-hydroxy-7,12-epoxycembra-1(2),3-diene.

**Table 2:** NMR data for compound **2**.

Position	*δ*_H_^a^ (*J* in Hz)	*δ*_C_^b^, type	HMBC	COSY	NOESY
1		151.0, qC			
2	6.05, d (8.4)	117.9, CH	14, 15		3, 15, 16, 18
3	6.37, d (8.4)	123.5, CH	5, 18	18	2, 7, 13a, 14a
4		132.6, qC			
5a	2.10, m	37.9, CH_2_	2	6a	6b
5b	2.27, m				
6a	2.04, m	30.8, CH_2_		5a, 7	
6b	1.84, m			7	5a, 19
7	4.05, t (3.2)	75.3, CH		6a, 6b	3, 9a, 13a
8		87.0, qC			
9a	2.13, m	36.6, CH_2_			7, 11
9b	2.09, m			10a	
10a	1.74 m	29.2, CH_2_		9b	
10b	1.08, m			11	19, 20
11	3.16, dd (10.0, 2.8)	78.3, CH		10b	9a, 10a, 13a
12		80.4, qC			
13a	1.97, m	37.4, CH_2_	12	14a	3, 7, 11
13b	1.82, m			14b	
14a	2.64, m	24.3, CH_2_	1	13a	3
14b	1.81, m		1	13b	
15	2.32, m	36.9, CH	1, 16, 17		2, 16, 17
16	1.06, d (6.8)	21.5, CH_3_	1, 15, 17	15	2, 15
17	1.04, d (6.8)	21.8, CH_3_	1, 15, 16	15	15
18	1.76, s	17.9, CH_3_	3, 4, 5	3	2
19	1.48, s	18.9, CH_3_	7, 8, 9		6b, 10b
20	1.11, s	18.1, CH_3_	11, 12, 13		10b, 14b
21		169.8,qC			
OAc	1.66, s	22.1, CH_3_	21		

^a^ Spectra were measured in C_6_D_6_ (400 MHz); ^b^ Spectra were measured in C_6_D_6_ (100 MHz).

The cytotoxicities of compounds **1** and **2** against P-388 (mouse lymphocytic leukemia), HT-29 (human colon adenocarcinoma) tumor cells, and human embryonic lung (HEL) cells are shown in Table 3. Compounds **1** and **2** were also examined for antiviral activity against human cytomegalovirus (HCMV) using a human embryonic lung (HEL) and displayed antiviral activity against human cytomegalovirus, with EC_50s_ of 20 and 8.0 µg/mL, respectively.

**Table 3:** Cytotoxicity and anti-HCMV activity of **1** and **2**.

Compounds	EC_50_ (µg/mL)				
	A549	HT-29	P-388	HEL	Anti-HCMV
**1**	>50	>50	25.9	>50	20
**2**	>50	>50	24.7	>50	8.0

## 3. Experimental Section

### 3.1. General Experimental Procedures

Optical rotations were determined with a JASCO P1020 digital polarimeter. UV and IR spectra were obtained on JASCO V-650 and JASCO FT/IR-4100 spectrophotometers, respectively. NMR spectra were recorded on a Varian MR 400 NMR spectrometer at 400 MHz for ^1^H and 100 MHz for ^13^C. ^1^H NMR chemical shifts are expressed in *δ* (ppm) referring to the solvent peak *δ*_H_ 7.27 for CHCl_3_ or *δ*_H_ 7.15 for C_6_D_6_, and coupling constants are expressed in Hertz (Hz). ^13^C NMR chemical shifts are expressed in *δ* (ppm) referring to the solvent peak *δ*_C_ 77.0 for CDCl_3_ or *δ*_C_ 128.0 for C_6_D_6_. MS were recorded by a Bruker APEX II mass spectrometer. Silica gel 60 (Merck, Germany, 230–400 mesh) and LiChroprep RP-18 (Merck, 40–63 µm) were used for column chromatography. Precoated silica gel plates (Merck, Kieselgel 60 F_254_, 0.25 mm) and precoated RP-18 F_254s_ plates (Merck) were used for thin-layer chromatography (TLC) analysis. High-performance liquid chromatography (HPLC) was carried out using a Hitachi L-7100 pump equipped with a Hitachi L-7400 UV detector at 220 nm together with a semi-preparative reversed-phased column (Merck, Hibar LiChrospher RP-18e, 5 µm, 250 × 25 mm).

### 3.2. Biological Material

The soft coral *S. ehrenbergi* was collected by SCUBA at San-Hsian-Tai, Taitong County, Taiwan, in July 2009 at a depth of 6 m and stored in a freezer until extraction. The voucher specimen (ST-13) was identified by Professor Chang-Feng Dai, National Taiwan University and deposited at the Department of Marine Biotechnology and Resources, National Sun Yat-sen University, Taiwan.

### 3.3. Extraction and Isolation

A specimen of soft coral *S. ehrenbergi* (2.0 kg) was minced and extracted with acetone (4 × 2 L) at room temperature. The combined acetone extracts were then partitioned between H_2_O and EtOAc. The resulting EtOAc extract (23.8 g) was subjected to gravity silica gel 60 column chromatography (Si 60 CC) using *n*-hexane and *n*-hexane/EtOAc of increasing polarity, to give 20 fractions. Fraction 14 (2.0 g), eluted with *n*-hexane/EtOAc (10:1), was further subjected to Sephadex LH-20 (acetone) to give 7 subfractions. The fraction 14-2-2 (0.108 g), was further subjected to RP-18 flash column (MeOH/H_2_O, 60:40 to 100% MeOH) to give 5 fractions. A subfraction 14-2-2-4 (12.9 mg), was purified by RP-18 HPLC (MeOH/H_2_O, 85:15) to afford **2** (2.3 mg, 0.0012%).The fraction 19 (0.2 g), eluted with *n*-hexane/EtOAc (1:8), was further subjected to RP-18 flash column (MeOH/H_2_O, 50:50 to 100% MeOH) to give 6 fractions. The subfraction 19-1, eluted with MeOH/H_2_O (50:50), was purified by RP-18 HPLC (MeOH/H_2_O, 50:50) to afford **1** (2.1 mg, 0.001%).

Ehrenbergol C (**1**): White amorphous powder; 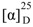 +95.0 (*c* 0.2, CHCl3); UV (MeOH) λ_max_ (log ε) 223 (3.42) nm; IR (neat) ν_max_ 3444, 2975, 2929, 1696, 1652, 1439, 1385, 1284, 1192, 1087, 1038, 754 cm^−^^1^; ^1^H NMR (CDCl_3_, 400 MHz) and ^13^C NMR (CDCl_3_, 100 MHz) data in Table 1; HRESIMS *m/z* 401.1939 [M + Na]^+^ (calcd for C21H30O6Na, 401.1940).

Acetyl ehrenberoxide B (**2**): White amorphous powder; 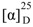 +25.0 (*c* 0.2, CHCl3); UV (MeOH) λ_max_ (log ε) 242 (3.2) nm; IR (neat) ν_max_ 3445, 2957, 2871, 1733, 1456, 1377, 1258, 1088, 1034, 772 cm^−^^1^; ^1^H NMR (C_6_D_6_, 400 MHz) and ^13^C NMR (C_6_D_6_, 100 MHz) data in Table 1; HRESIMS *m/z* 387.2512 [M + Na]^+^ (calcd for C22H36O4Na, 387.2511).

### 3.4. Cytotoxicity Assay

Cytotoxicity was determined on P-388 (mouse lymphocytic leukemia), HT-29 (human colon adenocarcinoma), and A-549 (human lung epithelial carcinoma) tumor cells using a modification of the MTT colorimetric method according to a previously described procedure [24,25,26]. The provision of the P-388 cell line was supported by J.M. Pezzuto, formerly of the Department of Medicinal Chemistry and Pharmacognosy, University of Illinois at Chicago. HT-29 and A-549 cell lines were purchased from the American Type Culture Collection. To measure the cytotoxic activities of tested compounds, five concentrations with three replications were performed on each cell line. Mithramycin was used as a positive control.

### 3.5. Anti-HCMV Assay

To determine the effects of natural products upon HCMV cytopathic effect (CPE), confluent human embryonic lung (HEL) cells grown in 24-well plates were incubated for 1 h in the presence or absence of various concentrations of tested natural products with three replications. Ganciclovir was used as a positive control. Then, cells were infected with HCMV at an input of 1000 pfu (plaque forming units) per well of a 24-well dish. Antiviral activity was expressed as IC_50_ (50% inhibitory concentration), or compound concentration required to reduce virus induced CPE by 50% after 7 days as compared with the untreated control. To monitor the cell growth upon treating with natural products, an MTT-colorimetric assay was employed [26,27,28].

## 4. Conclusions

This investigation of Taiwanese soft coral *S. ehrenbergi* collected has led to the isolation of two new ehrenbergol C and acetyl ehrenberoxide B (**1** and **2**). The carbon framework of **1** was identical to a cytotoxic diterpene, lobocrasol isolated from soft coral *Lobophytum crassum*. However, the stereochemistry of C-7 and C-8 of **1** were different from lobocrasol. Compounds **1** and **2** were not cytotoxic towards P-388 (mouse lymphocytic leukemia), HT-29 (human colon adenocarcinoma) tumor cells, and human embryonic lung (HEL) cells. However, compounds **1** and **2** displayed antiviral activity towards human cytomegalovirus, with IC_50_ of 20 and 8.0 μg/mL, respectively.

## Supplementary Files

Supplementary File 1 Supplementary Materials (PDF, 663 KB)
