# Supplementary material for: New Diterpenoids from Soft Coral Sarcophyton ehrenbergi
Source: Mar Drugs. 2013 Oct 30;11(11):4318–27. doi: 10.3390/md11114318 (PMC3853730; doi:10.3390/md11114318)

Supplementary Materials

Figure S1. ^{1}H NMR spectrum (400 MHz) of compound **1** in $CDCl_3$.

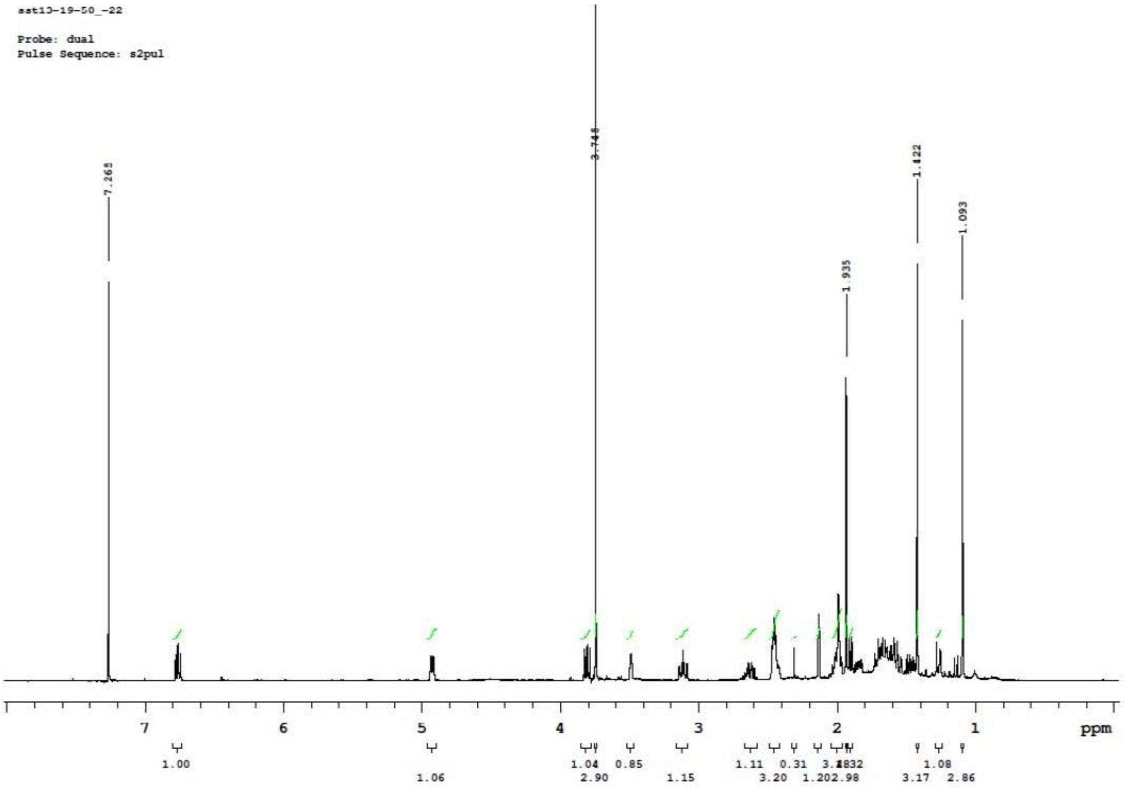

Figure S2. ^{13}C NMR spectra (100 MHz) of compound **1** in $CDCl_3$.

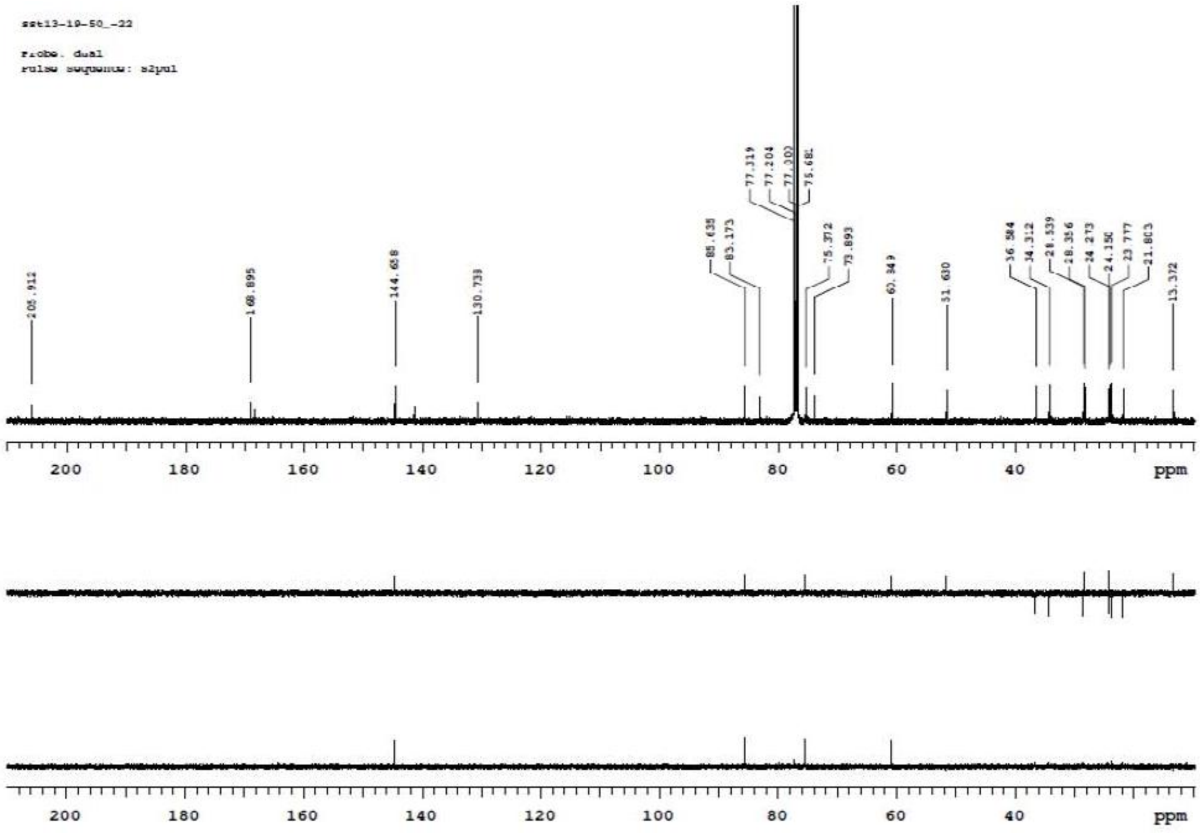

Figure S3. COSY spectrum (400 MHz) of compound **1** in $CDCl_3$.

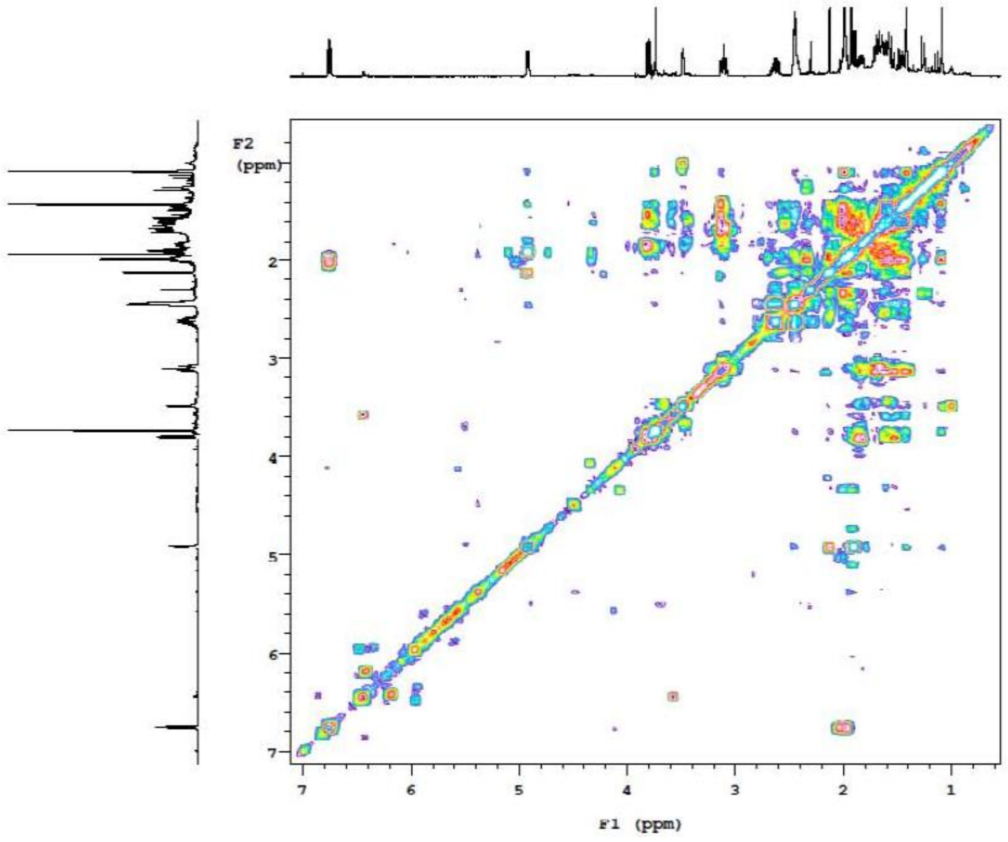

Figure S4. HSQC spectrum (400 MHz) of compound **1** in $CDCl_3$.

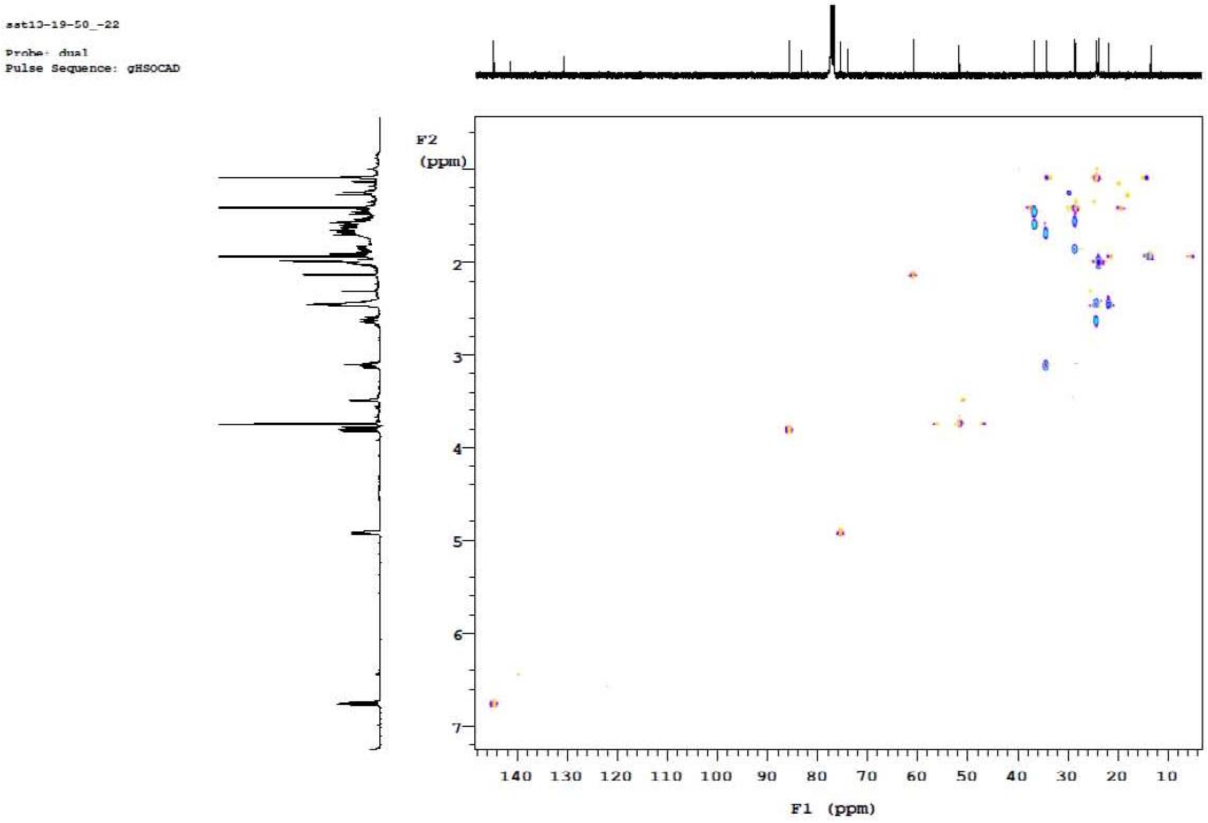

Figure S5. HMBC spectrum (400 MHz) of compound **1** in $CDCl_3$.

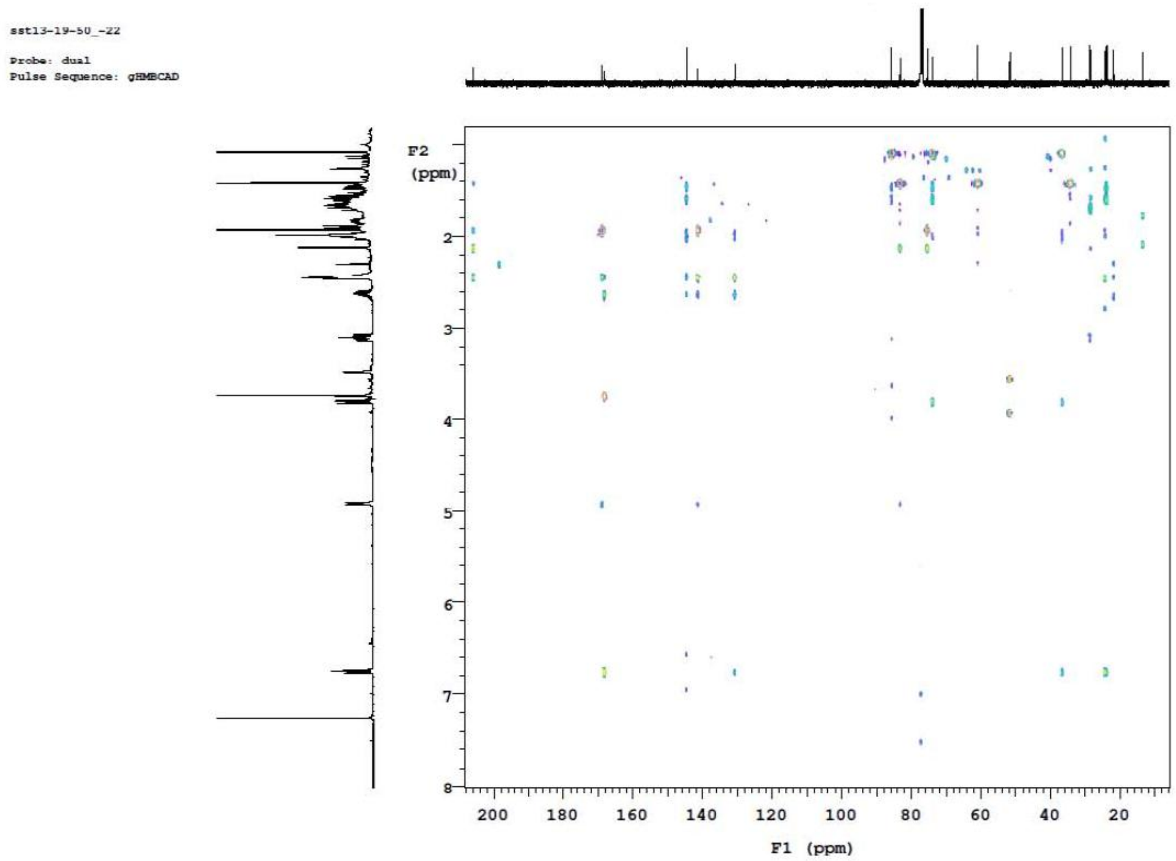

Figure S6. NOESY spectrum (400 MHz) of compound **1** in $CDCl_3$.

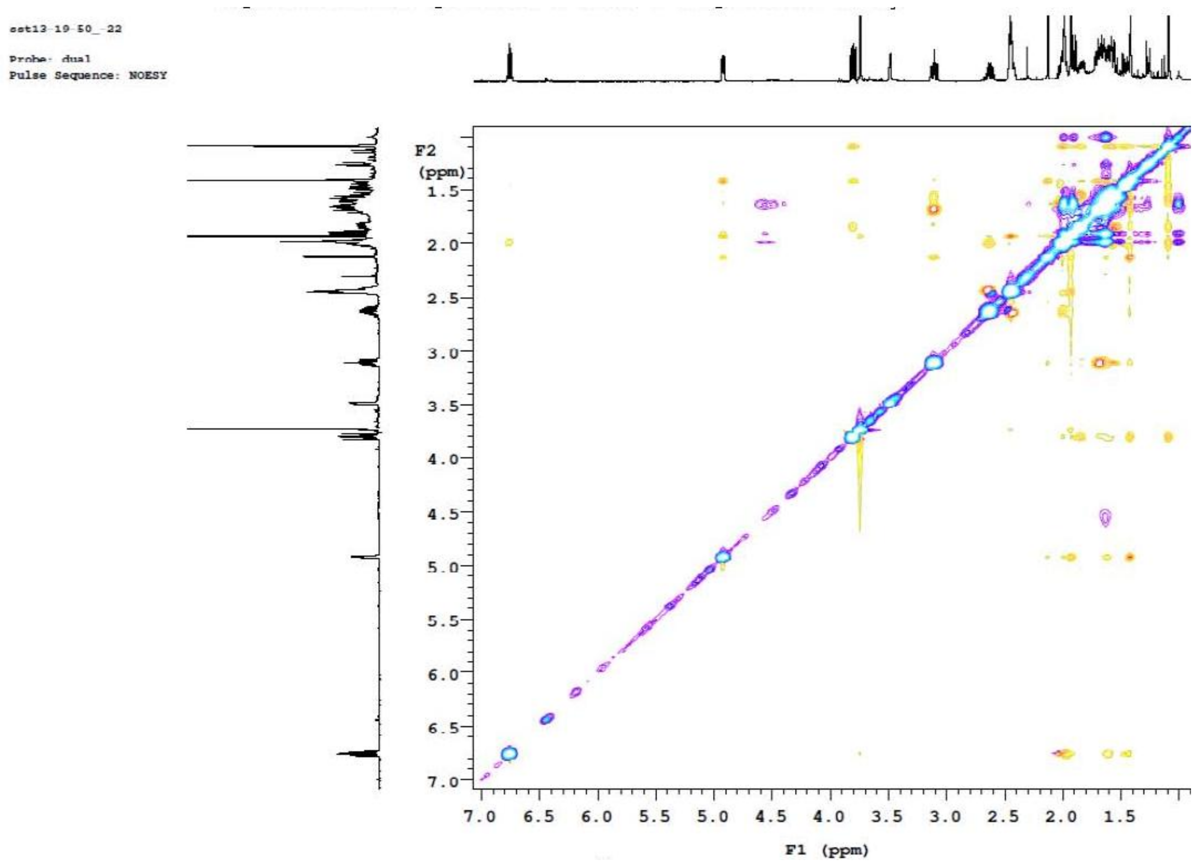

Figure S7. ^{1}H NMR spectrum (400 MHz) of compound **2** in C_6D_6.

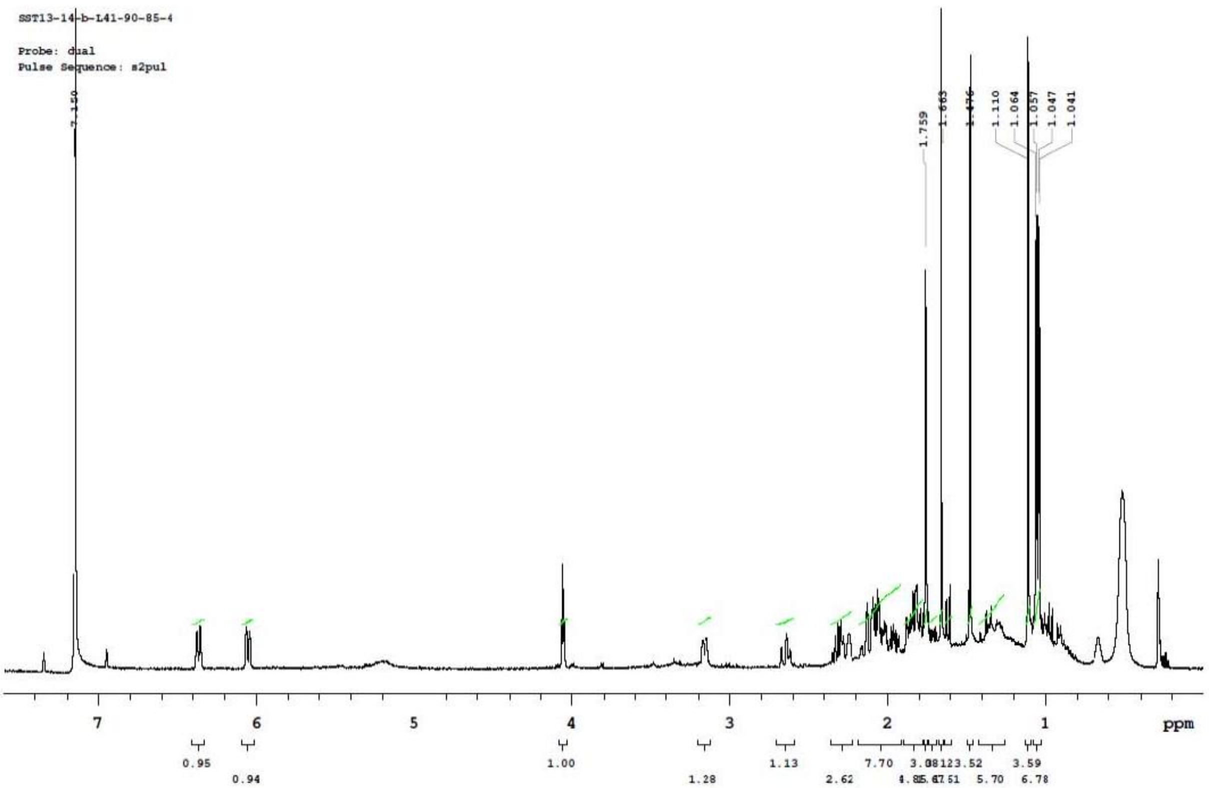

Figure S8. ^{13}C NMR spectra (100 MHz) of compound **2** in C_6D_6.

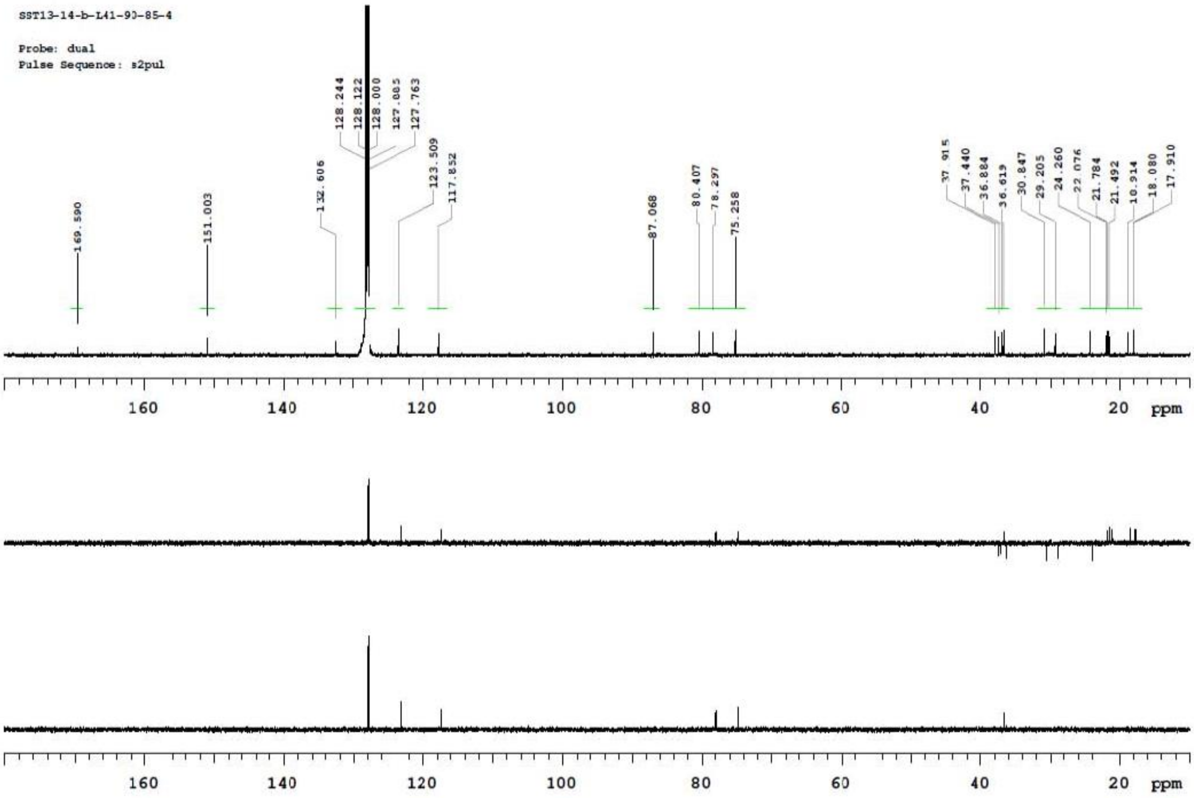

Figure S9. COSY spectrum (400 MHz) of compound **2** in C_6D_6.

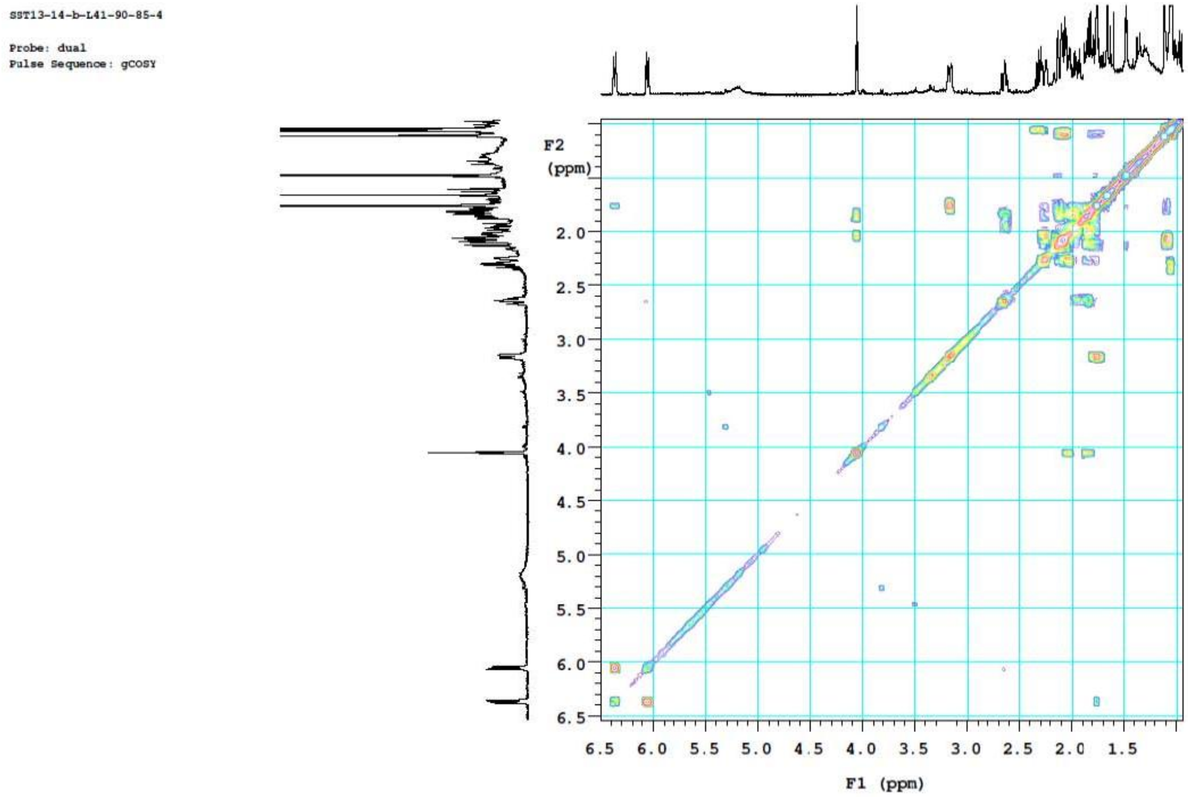

Figure S10. HSQC spectrum (400 MHz) of compound **2** in C_6D_6.

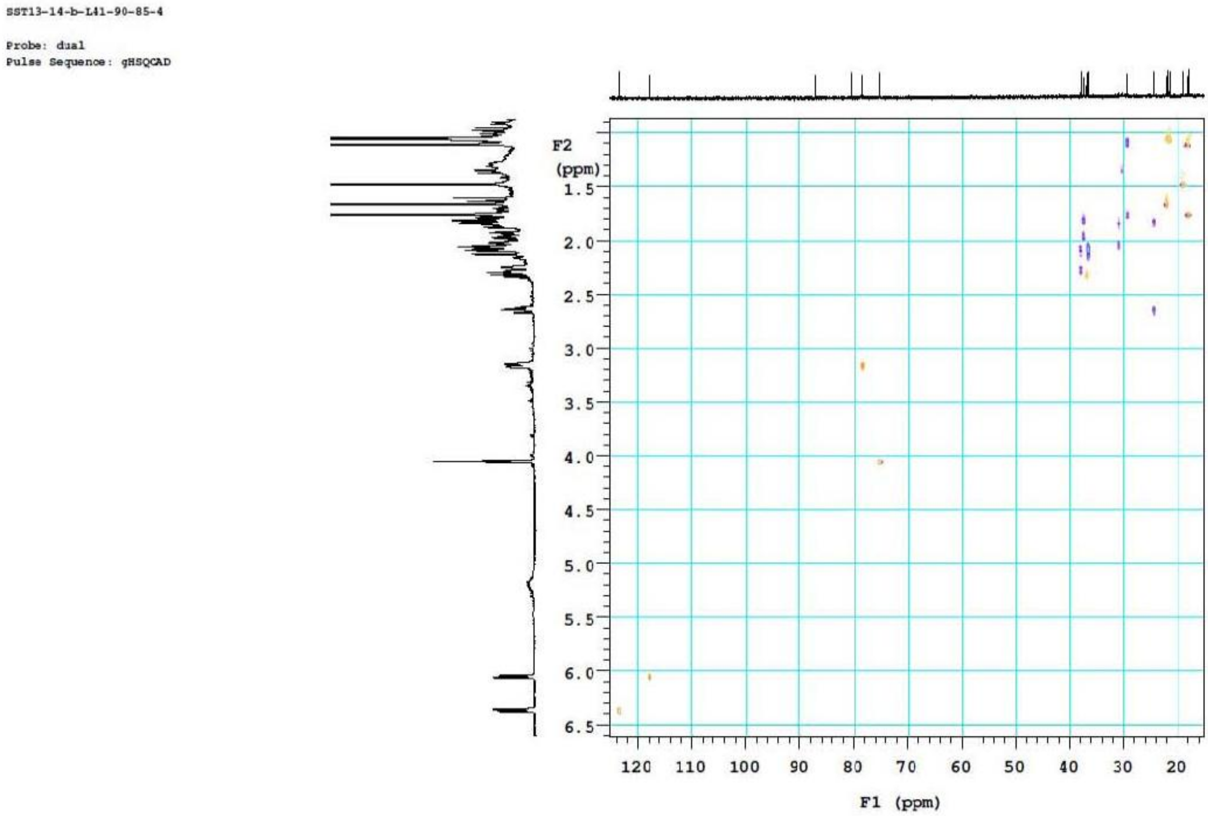

Figure S11. HMBC spectrum (400 MHz) of compound **2** in C_6D_6.

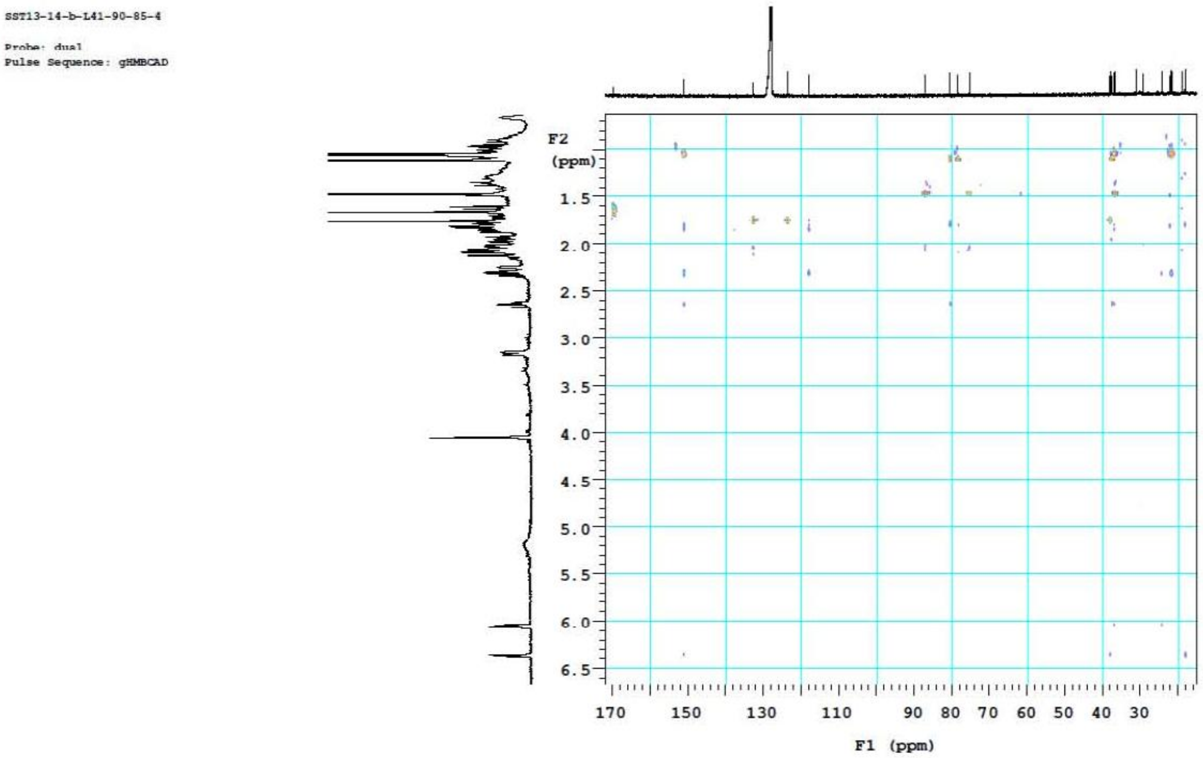

Figure S12. NOESY spectrum (400 MHz) of compound **2** in C_6D_6.

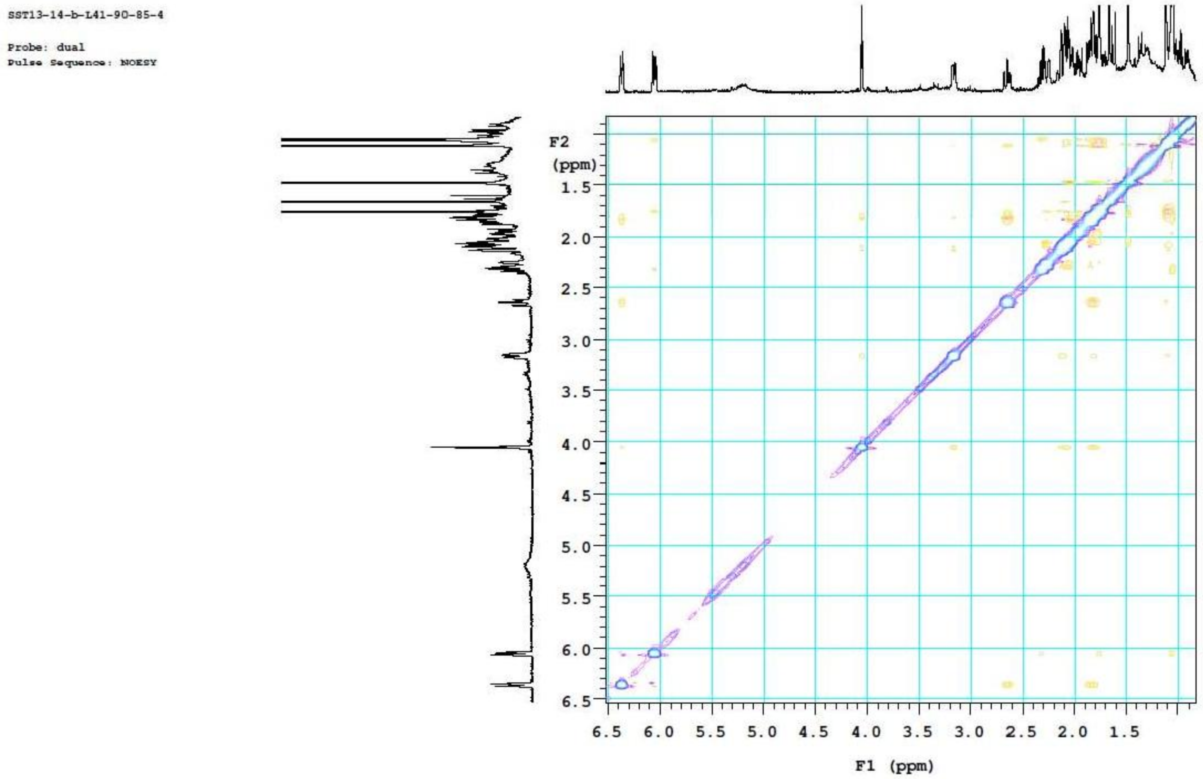

Supplement: Supplementary File 1 — Supplementary Materials (PDF, 663 KB) [file marinedrugs-11-04318-s001.pdf]
